# A rare case with multiple arterial variations of the liver complicated laparoscopic pancreaticoduodenectomy

**DOI:** 10.1186/s12876-022-02398-4

**Published:** 2022-07-07

**Authors:** Yong Yan, Bailin Wang, Wei Yuan, Jiansong Zhang, Junhao Xiao, Yanhua Sha

**Affiliations:** 1 Department of General Surgery, Guangzhou Red Cross Hospital, Jinan University, Guangzhou, 510220 China; 2 The Medical College of Guizhou Medical University, Guiyang, 550025 China; 3 Department of Laboratory Medicine, The Second Affiliated Hospital of Guangzhou University of Chinese Medicine, Guangzhou, 510120 China

**Keywords:** Anatomical variation, Replaced right hepatic artery, Middle hepatic artery, Replaced left hepatic artery, Laparoscopic pancreaticoduodenectomy, Case report

## Abstract

**Background:**

Hepatic arterial variations were fully elaborated in anatomical monographs. Here, we aimed to present a rare case with multiple arterial variations of the liver complicated laparoscopic pancreaticoduodenectomy.

**Case presentation:**

We report a 67-year-old woman with a periampullary tumor underwent laparoscopic pancreaticoduodenectomy. Intraoperatively, the aberrant right hepatic artery derived from the gastroduodenal artery (GDA) was observed and had accidentally sacrificed due to untimely ligature of GDA. Three-dimensional reconstruction based on preoperative contrast-enhanced CT performed to better study the anatomy. It demonstrated a replaced right hepatic artery branched from the GDA and supplied right liver lobe. Meanwhile, the middle hepatic artery derived from the common hepatic artery and supplied hepatic segment IV. Additionally, the replaced left hepatic artery emerged from the left gastric artery and fed into left liver lobe.

**Conclusions:**

The origination and course of hepatic arterial anatomy should be thoroughly assessed in planning and performing hepatopancreatobiliary surgeries. Reconstruction images of contrast-enhanced CT are helpful to visualize the vascular variations and its spatial relation with adjacent structures.

## Background

A great diversity of hepatic arterial variations were fully elaborated in anatomical monographs and frequently presented in case reports, which plays a crucial role in successful procedure of all hepatopancreatobiliary surgeries. As the standard anatomy of hepatic artery, the common hepatic artery (CHA) generally branches from the celiac trunk and mostly bifurcates into the proper hepatic artery (PHA) and the gastroduodenal artery (GDA). The PHA most commonly divides into the left hepatic artery (LHA), right hepatic artery (RHA), and sometimes middle hepatic artery (MHA) [1, 2]. The internationally accepted and widely applied classification method about hepatic arterial variations was devised by Michels [3] and subsequently further revised by Hiatt et al. [4]. The conventional arterial pattern of liver has been reported in 55 to 70% of subjects in studies with different detection methods, while various variations in hepatic arterial anatomy are seen in around one-third of the population [5]. One of the commonest anatomical arterial variations of liver is a RHA replaced from or an accessory RHA derived from the superior mesenteric artery (SMA) [6]. In this case report, we present a rare case with multiple arterial variations of the liver and discuss its surgical implications.

## Case presentation

**Fig. 1:**
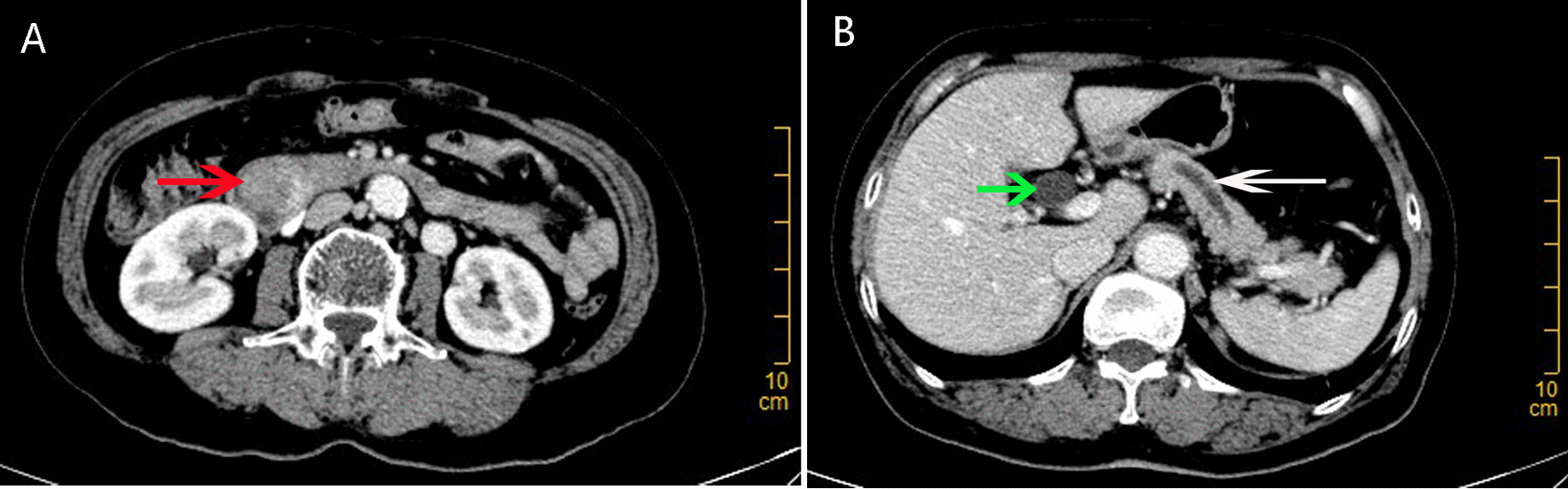
Abdominal contrast-enhanced CT revealed a hype-vascular tumor and dilation of the extrahepatic bile duct and pancreatic duct. **A** Axial CT image in the arterial stage showed a hype-vascular tumor (red arrow) located in the descending part of duodenum; **B** Axial CT image in the arterial stage showed dilation of the extrahepatic bile duct (green arrow) and pancreatic duct (white arrow)

A 67-year-old woman admitted to the nearby grass-roots hospital with a chief complaint of epigastric pain for 2 weeks, symptom was especially worse after meals. Routine abdominal computed tomography (CT) uncovered a neoplasm near the duodenal papilla associated with significant dilation of biliopancreatic duct. Then the woman was transferred to our tertiary hospital for further examination and therapy. The woman had no smoking history and did not drink alcohol, and was no significant history of hepatobiliary diseases, pancreatitis or abdominal operations. Physical examination was unremarkable, and without significant icterus. Blood routine test, renal function, electrolytes, coagulation function, and amylase were in the normal ranges. Liver function tests also were in the normal ranges with albumin 40.6 g/L, alanine aminotransferase 15 U/L, aspartate aminotransferase 25 U/L, gamma-glutamyltranspeptidase 31 U/L, total bilirubin 12.9 umol/L, and direct bilirubin 3.7 umol/L. Tumor markers revealed as following: alpha-fetoproteins 1.9 IU/mL, carcinoembryonic antigen 7.7 ug/L, carbohydrate antigen 19–9 1.0 U/mL. Abdominal contrast-enhanced CT showed a tumor located in the descending part of duodenum, the tumor measured about 3.7 cm in maximal diameter and possessed abundant vascularity (Fig. 1A). The extrahepatic bile duct and main pancreatic duct were significantly dilated with stricture at the site of duodenal papilla (Fig. 1B). The diagnosis of malignant periampullary tumor was confirmed by gastroscopic biopsy.

**Fig. 2:**
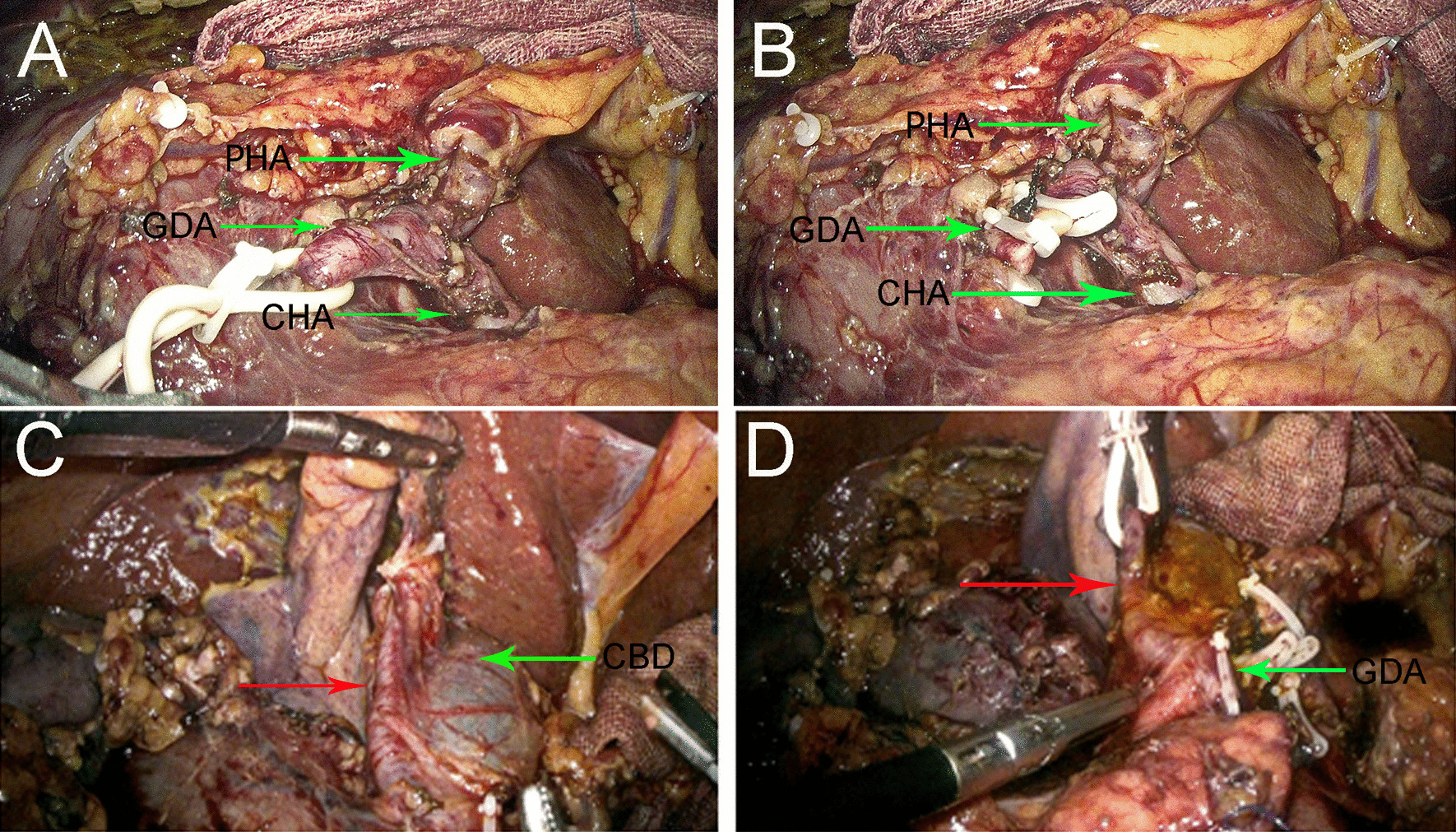
Intraoperative finding of a rare variation of hepatic artery derived from the GDA. **A** The CHA gave of two branches at superior border of pancreatic head, the one rose medial to CBD and extended to porta hepatis was regarded as PHA, and the other descended along pancreatic head was regarded as GDA. **B** The GDA was ligated at the origin from CHA. **C** A rare variation of hepatic artery (red arrow) derived from the GDA, passed in front of the CBD, distributed into the right liver lobe and was considered an aberrant RHA. **D** The aberrant RHA (red arrow) had accidentally sacrificed due to untimely ligature of GDA. *PHA* proper hepatic artery, *GDA* gastroduodenal artery, *CHA* common hepatic artery, *CBD* common bile duct, *RHA* right hepatic artery

The patient underwent laparoscopic pancreaticoduodenectomy (LPD) for radical resection of the malignant periampullary tumor. During dissection of the CHA, we recognized the CHA gave of two branches at superior border of pancreatic head (Fig. 2A). Habitually, the one was identified to be rising medial to common bile duct (CBD) and extended to porta hepatis and was regarded as PHA, as well as the other was identified to be descending along pancreatic head and was regarded as GDA. Since the root of GDA and PHA was exposed and isolated, the GDA was usually doubly ligated with suture and ligature clamp at its origin from CHA (Fig. 2B). Unexpectedly, a rare variation of hepatic artery derived from the GDA was observed after dissection of portal triad and considered an aberrant RHA, which arose in front of the CBD and entered into the right lobe of liver (Fig. 2C). Unfortunately, the aberrant RHA had accidentally sacrificed due to untimely ligature of GDA (Fig. 2D). At this stage, the aberrant RHA was considered an accessory RHA without reconstruction. Postoperative maximal values of alanine aminotransferase, aspartate aminotransferase, and total bilirubin value were 121 U/L on 7th postoperative day, 118 U/L on 1st postoperative day, and 49.7 umol/L on 1st postoperative day, respectively. The patient was discharged on the 14th postoperative day with gradually restored liver enzymes. The postoperative histopathologic examination revealed a periampullary adenocarcinoma. The postoperative follow-up was favorable with no distant metastasis and recurrence of tumor, as well as the liver function tests were with normal ranges during the 1-year follow-up.

**Fig. 3:**
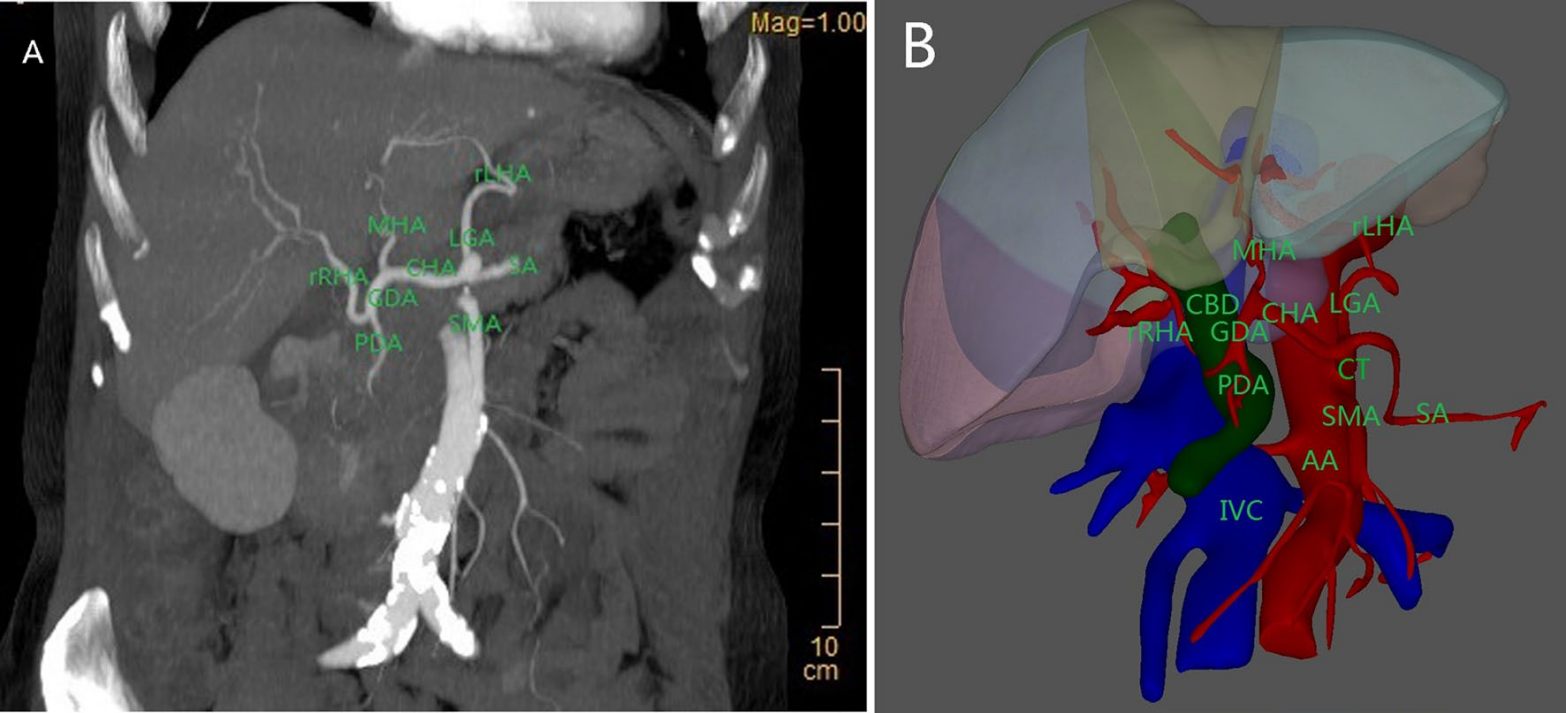
Coronal maximum intensity projection (MIP) and three-dimensional reconstruction images based on preoperative contrast-enhanced CT. **A** MIP image shows that the rRHA arose from the GDA, entered into the right liver lobe; MHA was derived from the CHA, and rLHA was branched from the LGA. **B** Three-dimensional reconstruction shows the spatial anatomical relationship of the liver, artery, and CBD; the rRHA traversed anterior to the CBD and supplied right liver lobe; the MHA extended to porta hepatis was erroneously identified as PHA intraoperatively, which entered the liver substance at the fissure between the right and left lobes, and actually supplied hepatic segment IV. *rRHA* replaced right hepatic artery, *rLHA* replaced left hepatic artery, *MHA* middle hepatic artery, *GDA* gastroduodenal artery, *LGA* left gastric artery, *SA* splenic artery, *PDA* pancreaticoduodenal artery, *CHA* common hepatic artery, *SMA* superior mesenteric artery, *CBD* common bile duct, *AA* abdominal aorta, *IVC* inferior vena cava, *CT* celiac trunk

Coronal maximum intensity projection and three-dimensional reconstruction images based on preoperative contrast-enhanced CT were performed to better assess arterial variations (Fig. 3). Reconstruction images showed the CHA originated from the celiac trunk as in classical anatomy. However, it was found that the CHA terminated as the GDA and middle hepatic artery (MHA). The MHA extended to porta hepatis was erroneously regarded as PHA intraoperatively, which entered the liver substance at the fissure between the right and left lobes, and actually supplied hepatic segment IV. There was no terminal branch from the CHA distributed to the right and left liver lobes. The GDA branched into the replaced RHA and superior pancreaticoduodenal artery in front of the pancreatic head about 1 cm away from its origin (Fig. 3A). The replaced RHA traversed anterior to the CBD and supplied right liver lobe. Additionally, the replaced LHA emerged from the left gastric artery (LGA) and fed into the left lobe of liver (Fig. 3B).

## Discussion and conclusions

Arterial anatomical variations of the liver occur due to the anomalous embryological development of the primitive ventral arteries from the dorsal aorta [7]. Michels [3] studied arterial anatomical variations of liver in 200 cadavers and set the influential benchmark for classification of hepatic artery variations in 1966, which initially classified 10 categories of the hepatic artery pattern (Table 1). Since his work there has been further investigation and modifications on the classification of these variations. Hiatt et al. [4] further summarized and modified this into six types (Table 2). Except for extremely rare variations of hepatic artery, many subsequent researches mostly employed these two classification systems to study arterial anatomical variations of liver. A literature review by Noussios et al. identified 20 articles and analyzed 19,013 cases of hepatic artery anatomy, which summarized that 81% of cases had classical hepatic artery anatomy that could be classified as Michels' classification of type I, and 15% of cases had variant hepatic artery pattern that could be classified as Michels' classification of type II–X [8]. From Noussios’s review of literature, the most common arterial anatomical variations of liver are the replaced RHA emerging from the SMA and the LGA giving off the accessory LHA, reported to occur in about 3.7% and 3.2%, respectively. Replaced RHA and accessory RHA were the most commonly arose from SMA (12.9%), followed by celiac trunk (0.24%), abdominal aorta (0.15%), and CHA (0.09%) [9]. In the present case, there was a replaced RHA emerged from the GDA. In addition, it was accompanied by MHA originated from the CHA and replaced LHA arose from the LGA. Although we were able to find two references to a RHA replaced from the GDA with incidence of less than 0.1% [10, 11], we could not find a report of a replaced RHA derived from the GDA accompanied by MHA originating from the CHA and replaced LHA originating from the LGA as in our case.

**Table 1:** Michels' classification of hepatic artery variations

Type	Description	Percent
I	Classic anatomy	55
II	rLHA arising from LGA	10
III	rRHA arising from SMA	11
IV	Coexistence of Type II and III	1
V	aLHA arising from LGA	8
VI	aRHA arising from SMA	7
VII	Coexistence of Type V and VI	1
VIII	rRHA from SMA + aLHA from LGA or aRHA from SMA + rLHA from LGA	2
IX	CHA arising from SMA	2.5
X	CHA arising from the LGA	0.5

*LHA* left hepatic artery, *RHA* right hepatic artery, *aLHA* accessory left hepatic artery, *aRHA* accessory right hepatic artery, *rLHA* replaced left hepatic artery, *rRHA* replaced right hepatic artery, *SMA* superior mesenteric artery, *CHA* common hepatic artery, *LGA* left gastric artery

**Table 2:** Hiatt’s classification of hepatic artery variations

Type	Description	Percent
I	Classic anatomy	75.7
II	rLHA or aLHA arising from LGA	9.7
III	rRHA or aRHA arising from SMA	10.6
IV	Coexistence of Type II and III	2.3
V	CHA arising from SMA	1.5
VI	CHA arising from aorta	0.2

*aLHA* accessory left hepatic artery, *aRHA* accessory right hepatic artery, *rLHA* replaced left hepatic artery, *rRHA* replaced right hepatic artery, *SMA* superior mesenteric artery, *CHA* common hepatic artery, *LGA* left gastric artery

In general, it was defined as a MHA when there was a dominant hilar artery supplied segment IV of the liver. The MHA can arise from the trunk and branches of the RHA, LHA, or PHA [12]. Wang et al. [13] reported an MHA was present in 71% of the living subjects detected by contrast-enhanced CT, and summarily classified the anatomical variations of MHA into five types according to the origination of MHA. They reported that MHA always branched directly or indirectly from the CHA, from which the GDA also originated. In a study evaluated with 455 potential donors by multi-detector CT, Nazer et al. [14] showed that MHA was emerging mostly from LHA (62.6%) followed by RHA (24.4%), CHA (6.4%), PHA (1.8%), and even GDA (0.7%). The replaced and accessory LHA derived from the LGA reported to occur in approximately 10% of the 1000 patients of liver donor studied by Hiatt et al. [4]. In Cirocchi’s [15] systematic review of 57 studies and 19,284 cases of left hepatic artery anatomy, the overall prevalence of a anomalous LHA derived from LGA was about 13%, included 8% of cases had replaced LHA and 5% of cases had accessory LHA. In our case, there were combined variations of MHA emerged from CHA and replaced LHA emerged from the LGA.

Now there are still lots of debates on whether it is feasible to ligate aberrant hepatic artery without reconstruction during abdominal surgeries. Since the early 20th century, some researches have revealed that there were no connecting arteries between RHA and LHA within the liver in cadaveric studies, so the hepatic artery was considered as an end artery [16]. Subsequently, studies of digital subtraction angiography in living subjects demonstrated that there also was sufficient blood flow to the relevant liver lobe although one of the hepatic arteries was ligated, which could be explained by the compensatory collateral circulation to the liver. The development of collateral circulation could be attributed to the neurohumoral factors that play an important role in opening up the existing extrahepatic pathways [17]. When a replaced hepatic artery is incorporated by tumors in patients undergoing pancreatoduodenectomy, either resection of the replaced hepatic artery with reconstruction or resection of the replaced hepatic artery without reconstruction is an important consideration. In some reports, replaced hepatic artery was sometimes sacrificed without reconstruction which could not lead to serious adverse events or compromised oncological outcomes [18]. Other articles described that resection the replaced hepatic artery could lead to severe morbidities such as liver necrosis and liver failure, which advised that the replaced hepatic artery should be reconstructed [19]. In a study observed ligation of aberrant LHA in 204 patients, Ang et al. [20] concluded that ligation of replaced LHA is safe as it only resulted in transient rise of liver enzymes. In contrast, another study stated that sacrificing the replaced LHA could increase the risk of hepatic ischemia, hepatic abscess formation, liver necrosis, and even liver failure [21]. Nevertheless, we considered that preserving, resection followed reconstruction and avoiding inadvertent injury of a replaced hepatic artery were important for decreasing the risk of liver atrophy and hepatic insufficiency, especially necessary for the patient with decompensated cirrhosis.

In our case, the patient underwent LPD for radical resection of the malignant periampullary tumor. During LPD, the CHA usually was dissected in the superior border of pancreatic head after division of the gastrocolic ligament and visualization of the anterior surface of the pancreas, the CHA most commonly gave of two branches at superior border of pancreatic head, one was PHA rose within the left of hepatoduodenal ligament and extended to the liver hilar, and the other was GDA descended along pancreatic head. Generally, the GDA was doubly ligated with suture and ligature clamp at the origin from CHA after the root of GDA and PHA was exposed and isolated. If unrecognized preoperatively, this rare variation of anomalous origin of the replaced RHA derived from GDA could be inadvertently sacrificed due to untimely ligature of GDA resulting in compromised blood flow of right liver lobe. Furthermore, the aberrant course of the replaced RHA rising anterior to the CBD could pose signifcant risk of iatrogenic arterial injury during the process of dissociation of porta hepatis, which could lead to hemorrhage, biliary leakage, or hepatic ischemia [22]. The GDA habitually is ligated at its proximal origination after take-off from the CHA in cases without hepatic arterial variation, which is an important step during pancreaticoduodenectomy. When preoperative contrast-enhanced CT or arteriographic images demonstrated arterial variations of a replaced RHA with abnormal origin and course, its pulse could be touched within the hepatoduodenal ligament intraoperatively during porta hepatis dissociation undergoing laparotomy surgery. If the origination was identified arising from the GDA as in this case, the GDA should be ligated distal to the origination after its bifurcation of the replaced RHA for the purpose of avoiding inadvertent arterial injury and preserving sufficient blood flow to the right lobe of liver [23]. In contrast to open surgery, laparoscopic procedures decrease tactile sense and add complexity for dissection along the blood vessels to throughly expose its course and origination. Taken together, encountering an unusually large GDA at pancreaticoduodenectomy should also alert the surgeon as to whether one is dealing with an artery that might be supplying the liver, even if the location and direction is suggestive of GDA.

In conclusion, the origination and course of hepatic arterial anatomy in each patient should be thoroughly assessed in planning and performing hepatopancreatobiliary surgeries to avoid vascular complications and decrease morbidities. Contrast-enhanced CT is an important method to find and identify variations of hepatic blood vessel. Reconstruction images of contrast-enhanced CT are helpful to visualize the vascular variations and its spatial relation with adjacent structures and facilitate surgical planning.

## Data Availability

All data generated or analyzed during this study are included in this published article.

## Abbreviations

LHA: Left hepatic artery
RHA: Right hepatic artery
aLHA: Accessory left hepatic artery
aRHA: Accessory right hepatic artery
rLHA: Replaced left hepatic artery
rRHA: Replaced right hepatic artery
MHA: Middle hepatic artery
SMA: Superior mesenteric artery
CHA: Common hepatic artery
LGA: Left gastric artery
CBD: Common bile duct
GDA: Gastroduodenal artery
PHA: Proper hepatic artery
SA: Splenic artery
PDA: Pancreaticoduodenal artery
AA: Abdominal aorta
IVC: Inferior vena cava
CT: Computer tomography
LPD: Laparoscopic pancreaticoduodenectomy
